# Primary adrenal non-Hodgkin lymphoma: a case report and review of the literature

**DOI:** 10.1186/s13256-017-1271-x

**Published:** 2017-04-15

**Authors:** Nanik Ram, Owais Rashid, Saad Farooq, Imran Ulhaq, Najmul Islam

**Affiliations:** 1 Department of Medicine, Section of Endocrinology, Aga Khan University, Karachi, Pakistan; 2 Aga Khan University, Stadium Road, Karachi, Pakistan

**Keywords:** Primary adrenal lymphoma, Non-Hodgkin lymphoma, Adrenal mass, Case report

## Abstract

**Background:**

Lymphomas are cancers that arise from the white blood cells and have been traditionally divided into two large subtypes: Hodgkin and non-Hodgkin lymphoma. B-cell lymphoma is the most common subtype of non-Hodgkin lymphoma; almost 85% of patients with lymphoma have this variant. Lymphomas can potentially arise from any lymphoid tissue located in the body; however, primary adrenal non-Hodgkin lymphoma is extremely rare. We report the history, examination findings, and laboratory results of a 50-year-old man diagnosed with a primary left adrenal diffuse large B-cell lymphoma.

**Case presentation:**

A 50-year-old Pakistani man presented to our hospital with progressively increasing pain and fullness in the left upper quadrant of his abdomen, generalized weakness, easy fatigability, and decreased appetite of 1.5 months’ duration. On examination, he had a blood pressure of 140/80 mmHg with no postural drop, a pulse rate of 106 beats/minute, and no fever. His past medical history was significant for pulmonary tuberculosis 2 years earlier, for which he received antituberculous therapy. Computed tomography revealed a heterogeneous enhancing soft tissue density mass in the left adrenal gland. It measured 7.1 × 5.6 × 9.5 cm. Further laboratory workup revealed the following levels: sodium 135 mEq/L, potassium 4.5 mEq/L, lactate dehydrogenase 905 IU/L, renin 364 IU/ml, aldosterone 5.79 ng/dl, dehydroepiandrosterone sulfate 79.20 μg/dl, urinary vanillylmandelic acid 6.4 mg/24 hours, and a low-dose overnight dexamethasone suppression test result of 3.20 μg/dl. The patient underwent left adrenalectomy. Histopathological test results showed a diffuse large B-cell lymphoma. Immunohistochemical stains were strongly positive for CD20 and negative for CD3, CD5, CD10, and cyclin D1. The patient’s Ki-67 (Mib-1) index was approximately 80%. He received a total of six cycles of cyclophosphamide, doxorubicin, vincristine, and prednisone chemotherapy (rituximab was not given, owing to financial constraints) and was routinely followed pre- and postchemotherapy at our hematology clinic with complete blood count and serum lactate dehydrogenase evaluations. The patient responded to chemotherapy and is currently doing well.

**Conclusions:**

Primary adrenal lymphoma is an extremely rare but rapidly progressive disease. It generally carries a poor prognosis, partly because an optimal treatment protocol has not yet been established. Further studies with larger sample sizes are needed to establish the best treatment option and increase overall survival.

## Background

The American Cancer Society estimated that more than 70,000 new cases of non-Hodgkin lymphoma (NHL) would be diagnosed in 2016 [1, 2]. Although lymphomas arise mainly from lymph nodes, primary extranodal NHL occurs in at least 25% of the cases [3]. The adrenal gland can be secondarily involved in around 4% of the patients; however, primary adrenal NHL is extremely rare and accounts for less than 1% of all NHL cases [4]. Primary adrenal lymphoma (PAL) is histologically proven lymphoma of one or both adrenal glands in patients with no prior history of lymphoma. If other organs or lymph nodes besides the adrenal glands are involved, the adrenal lesion must be unequivocally dominant [5].

PAL occurs predominantly in males in the sixth to seventh decades of life. Most commonly, this lymphoma is a nongerminal center-type diffuse large B-cell lymphoma (DLBCL), which is present in 70% to 80% of the patients [6]. Patients present with abdominal or lumbar pain; fever; weight loss; and signs of adrenal insufficiency such as hypotension, hyponatremia, fatigue, skin hyperpigmentation, and vomiting [7]. In occasional instances, it may also be an incidental finding on imaging studies obtained for other purposes, and it is frequently bilateral and bulky at the time of presentation. Several etiological factors, such as Epstein-Barr virus infection, genetic defects in p53 and c-kit, and immune dysregulation, have been implicated in the pathogenesis of this disease [5, 7]. Laboratory investigations often show elevated lactate dehydrogenase (LDH), β_2_-microglobulin, C-reactive protein, and ferritinemia, which signify high levels of inflammation associated with PAL [6].

The prognosis of this condition is generally considered to be poor because PAL is an aggressive disease and progresses rapidly. An average 1-year survival as low as 20% has been reported; however, owing to the rare nature of this disease, prognostic factors are difficult to elucidate [5].

## Case presentation

A 50-year-old Pakistani man known to have had diabetes for 21 years presented to our hospital with progressively increasing pain and fullness in the left upper quadrant of his abdomen, generalized weakness, easy fatigability, and decreased appetite of 1.5 months’ duration. He also complained of nausea and early satiety and had a weight loss of 8 kg over this period. On examination, he was found to have a blood pressure of 140/80 mmHg with no postural drop, a pulse rate of 106 beats/minute, and no fever. His physical examination was otherwise unremarkable. His past medical history was significant for pulmonary tuberculosis 2 years earlier, for which he received antituberculous therapy.

**Fig. 1:**
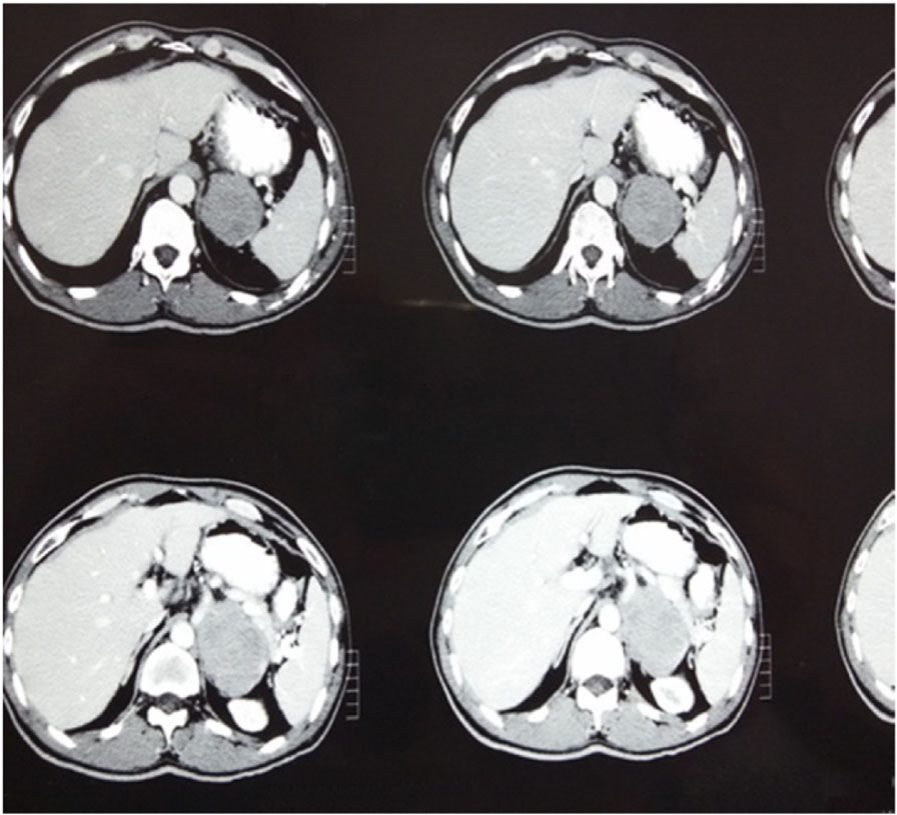
Computed tomography of the patient showing a large left adrenal mass

The patient had initially presented at another university hospital 3 weeks earlier. At that time, a laboratory workup and computed tomography (CT) of the abdomen with contrast enhancement were done. Although the results of his complete blood count and renal function test were normal, CT of the abdomen showed a heterogeneous enhancing soft tissue density mass in the left adrenal gland. The mass measured 7.1 × 5.6 cm in transverse and anteroposterior diameter, and the craniocaudal extent of the mass was 9.5 cm. Medially, the mass was abutting the celiac and superior mesenteric arteries, and posteroinferiorly, it was bordering the renal vessels. Paraaortic lymphadenopathy was also present, with the largest one measuring 1.6 cm (Fig. 1).

**Fig. 2:**
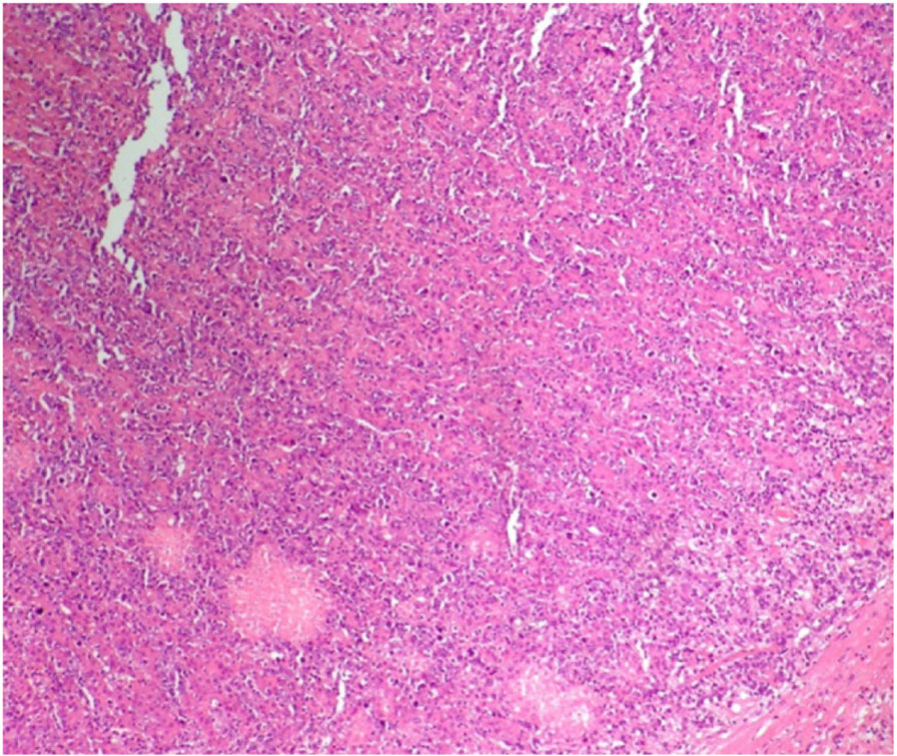
Low-power view of the lesion showing diffuse sheets of neoplastic cells (hematoxylin and eosin stain)

**Fig. 3:**
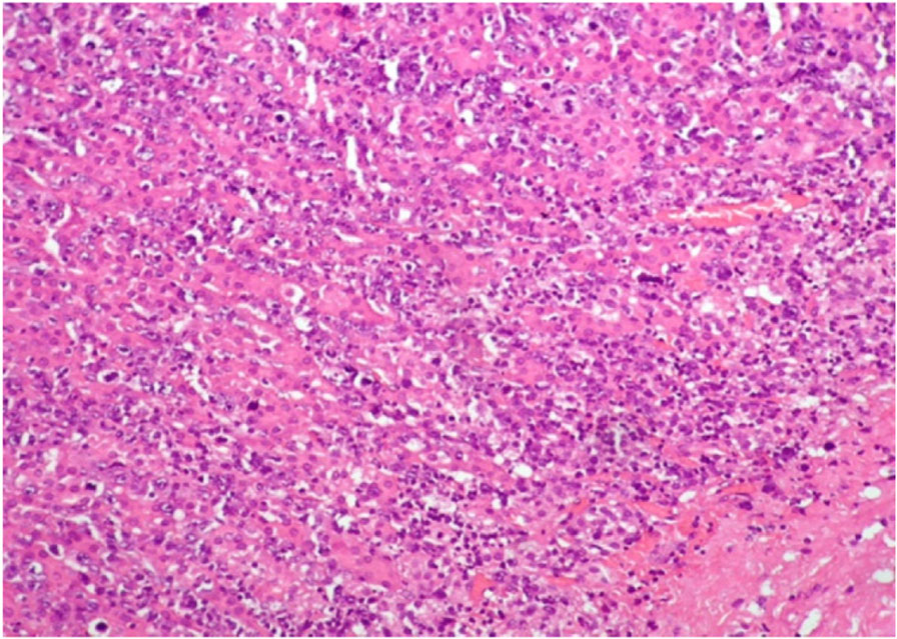
High-power view of the lesion showing large-sized neoplastic cells with pleomorphic nuclei, variably prominent nucleoli, and scant cytoplasm. Frequent mitotic figures are also noted (hematoxylin and eosin stain)

**Fig. 4:**
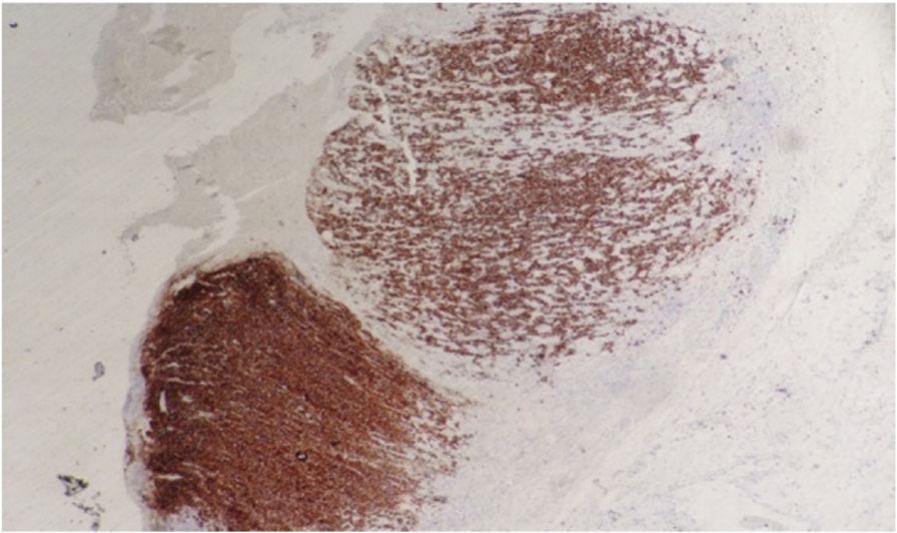
CD20 immunohistochemical stain (pan-B) is strongly positive for neoplastic cells

**Fig. 5:**
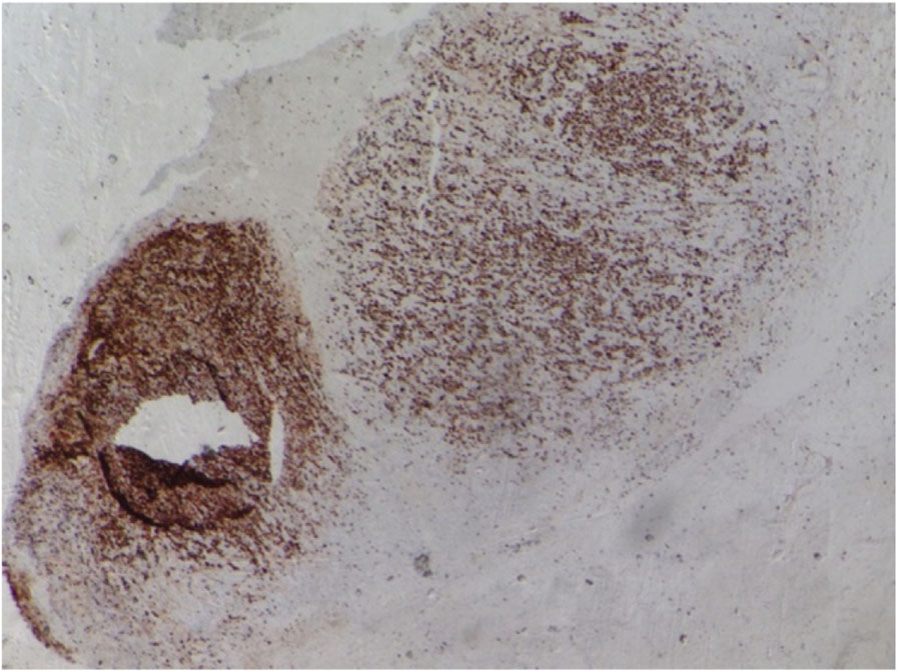
Ki-67 immunohistochemical stain highlights a markedly raised proliferative index in the neoplastic lymphoid population

Further laboratory workup revealed the following levels: sodium 135 mEq/L, potassium 4.5 mEq/L, LDH 905 IU/L, renin 364 IU/ml, aldosterone 5.79 ng/dl, dehydroepiandrosterone sulfate 79.20 μg/dl, urinary vanillylmandelic acid 6.4 mg/24 hours, and a low-dose overnight dexamethasone suppression test result of 3.20 μg/dl. The patient was referred to our urology clinic for surgical removal of his mass. He underwent a left adrenalectomy at the urology clinic on 4 March 2016. Histopathological analysis revealed DLBCL (Figs. 2 and 3). The results of immunohistochemical stains were strongly positive for CD20 and negative for CD3, CD5, CD10, and cyclin D1. His Ki-67 (Mib-1) index was approximately 80% (Figs. 4 and 5).

For further management, the patient was referred to our hematology clinic and was planned for a rituximab, cyclophosphamide, doxorubicin, vincristine, and prednisone (R-CHOP) chemotherapy regimen starting on 18 March 2016. He received a total of six cycles of cyclophosphamide, doxorubicin, vincristine, and prednisone (CHOP; rituximab was not given, owing to financial constraints) and was routinely followed pre- and postchemotherapy at the hematology clinic with complete blood count and serum LDH evaluations. Positron emission tomography (PET) performed on 24 March 2016 showed metabolically active residual disease over the left adrenal bed. Subcentimetric fluorodeoxyglucose (FDG) deposits were seen in the patient’s L5, L2, and T12 vertebrae, suggestive of marrow infiltration. No evidence of hypermetabolic nodal, hepatic, or splenic involvement was appreciated. However, the patient responded to chemotherapy and is currently doing well. He gained around 8 kg of weight and is following his routine daily activities. A recent PET scan revealed that the previously seen hypermetabolic foci along the left crus, left proximal paraaortic region, and foci of FDG uptake in the lumbar and thoracic vertebrae were not appreciable. The patient’s Deauville 5-point scale score was 0 (complete metabolic response).

## Discussion

To the best of our knowledge, this is the first report describing a case of primary adrenal NHL in Pakistan. Our patient was a man in his fifth decade of life, which, on the basis of published literature, is a relatively young age to have this disease. We treated our patient with a regimen of CHOP; rituximab was not included, owing to financial constraints. Even without rituximab, our patient showed a complete response to therapy. Because primary adrenal NHL is a rare disease, optimal treatment has not yet been established. CHOP or CHOP-like regimens were traditionally used before the introduction of rituximab, with generally dismal results (overall survival between 20% and 50%) [7]. In the largest study to date on PAL, involving 31 patients given an R-CHOP chemotherapy regimen, complete remission and overall response rates were 54.8% and 87.0%, respectively. Surprisingly, no difference was found in overall survival between unilateral and bilateral NHL of the adrenal gland [8]. Our patient also underwent adrenalectomy; however, the two largest studies to date showed no survival benefit in patients who underwent adrenalectomy as compared with those who were treated with chemotherapy alone [6, 8].

In another study involving 28 patients with PAL, 64% of the patients were treated with a CHOP regimen, 50% with an R-CHOP regimen, and 18% had chemotherapy with doxorubicin, cyclophosphamide, vindesine, bleomycin, and prednisone. The overall survival was 61.6%; however, it was 100% for those who received autologous stem cell transplants, which suggests that this may prolong survival [6]. The Ki-67 index was high in our patient (80%). Ichikawa *et al*. reported this index to be greater than 70% in seven patients with primary adrenal DLBCL. They treated all of these patients with rituximab-containing chemotherapy and reported a 2-year survival rate of 57%, although none of the patients died as a result of advancement of lymphoma [9]. These authors suggested that rituximab-containing chemotherapy with central nervous system (CNS) prophylaxis with methotrexate may be a good treatment option for primary adrenal NHL. Kim *et al*. reported CNS relapses or progression in four patients, none of whom had received intrathecal prophylaxis [8].

For our patient, we opted for CT as the initial imaging modality and then confirmed the diagnosis via histology of the resected adrenal gland. Grigg *et al*. suggested that though magnetic resonance imaging and CT findings can be highly suggestive of NHL, a biopsy should be done for diagnosis. Staging should involve a PET or gallium scan, and in patients with elevated LDH levels, a lumbar puncture should also be done [7].

According to the International Prognostic Index (IPI), our patient was in the low- to intermediate-risk category, which has an estimated 5-year survival of 51%. However, this scoring system is not specific to PAL; thus, it may be inaccurate in predicting overall survival. Kim *et al*. found that neither high-risk IPI score nor advanced-stage disease according to the Ann Arbor system had any impact on the overall survival; therefore, they suggested a modified IPI scoring system and a revised staging system, which resulted in significantly improved predictability of overall survival [9]. The modified scoring and staging criteria may prove beneficial in risk stratification of patients with primary adrenal NHL and also guide treatment.

No protocol for specific treatment in cases of a primary adrenal NHL has yet been established, and multiple authors have used a combination of modalities, including surgery and chemotherapy. Ichikawa *et al*. argued that perhaps one of the reasons for the poor prognosis is that many patients were previously treated with chemotherapy not containing rituximab and did not receive CNS prophylaxis, which may have decreased overall survival [9].

## Conclusions

PAL is a rare but rapidly progressing disease that should be treated aggressively. Rituximab-containing chemotherapy such as R-CHOP has shown promise by increasing the overall survival of patients with this disease. R-CHOP combined with CNS prophylaxis and autologous stem cell transplant may further increase overall survival, but further studies with larger sample sizes are needed to establish the best treatment option and decide whether surgery and radiation have a role in the management of PAL.

## Abbreviations

CHOP: Cyclophosphamide, doxorubicin, vincristine, and prednisone
CNS: Central nervous system
CT: Computed tomography
DLBCL: Diffuse large B-cell lymphoma
FDG: Fluorodeoxyglucose
IPI: International Prognostic Index
LDH: Lactate dehydrogenase
NHL: Non-Hodgkin lymphoma
PAL: Primary adrenal lymphoma
PET: Positron emission tomography
R-CHOP: Rituximab, cyclophosphamide, doxorubicin, vincristine, and prednisone
